# 3D-Slicer Software-Assisted Neuroendoscopic Surgery in the Treatment of Hypertensive Cerebral Hemorrhage

**DOI:** 10.1155/2022/7156598

**Published:** 2022-02-18

**Authors:** Rongfang Liao, Longmao Liu, Bo Song, Xinhong Wan, Shuo Wang, Jianhong Xu

**Affiliations:** The Second People's Hospital of Jingdezhen City, Jiangxi Province 333000, China

## Abstract

**Objective:**

To explore the 3D-slicer software-assisted endoscopic treatment for patients with hypertensive cerebral hemorrhage.

**Methods:**

A total of 120 patients with hypertensive cerebral hemorrhage were selected and randomly divided into control group and 3D-slicer group with 60 cases each. Patients in the control group underwent traditional imaging positioning craniotomy, and patients in the 3D-slicer group underwent 3D-slicer followed by precision puncture treatment. In this paper, we evaluate the hematoma clearance rate, nerve function, ability of daily living, complication rate, and prognosis.

**Results:**

The 3D-slicer group is better than the control group in various indicators. Compared with the control group, the 3D-slicer group has lower complications, slightly higher hematoma clearance rate, and better recovery of nerve function and daily living ability before and after surgery. The incidence of poor prognosis is low.

**Conclusion:**

The 3D-slicer software-assisted endoscopic treatment for patients with hypertensive intracerebral hemorrhage has a better hematoma clearance effect, which is beneficial to the patient's early recovery and reduces the damage to the brain nerve of the patient.

## 1. Introduction

Hypertensive intracerebral hemorrhage [1–4] is the most serious complication in the development of hypertensive disease. It has a high disability rate and fatality rate. This disease is very common in neurosurgery and has a great impact on the patient's quality of life and ability of daily living [5–7]. Relevant data point out that the incidence of men is higher than that of women and the patients are in poor mood.

Nowadays, the population is seriously aging, and the number of patients with hypertensive cerebral hemorrhage is gradually increasing. Surgical treatment is often used. In the past, the operation was a craniotomy, which was not effective. It is prone to infection and rebleeding after the operation. With the improvement of medical technology, the 3D-slicer software [8–11] is gradually improved and has a higher clinical application rate. Compared with craniotomy, the use of 3D-slicer software to assist endoscopic treatment has the characteristics of simple operation and high safety [12–15].

At present, domestic neuroendoscopic surgery for hypertensive cerebral hemorrhage mostly uses traditional body surface projection method to locate, but it is often difficult to accurately grasp the width and depth of the hematoma, which affects the effect of the operation. The 3D-slicer is an open source software that has attracted much attention in surgical auxiliary applications in recent years [16]. The software can perform three-dimensional reconstruction of hematoma before surgery, calculate the volume of hematoma, and design surgery through virtual reality and augmented reality technology. The puncture path plays a better auxiliary positioning value.

With the continuous development of imaging examination technology and surgical technology, clinically based on the clinical characteristics of the patient, head computed tomography (CT) [17–19] is used to check the location, size, degree of edema of the surrounding brain tissue, and whether there is cerebral edema. We rely on the results of the examination to remove the hematoma from the patient [20]. In this study, the effect of precision puncture treatment of hypertensive cerebral hemorrhage with 3D-slicer software was analyzed in order to improve the prognosis of patients and improve the treatment effect.

## 2. Methods and Materials

### 2.1. Structure of This Study

**Figure 1:**
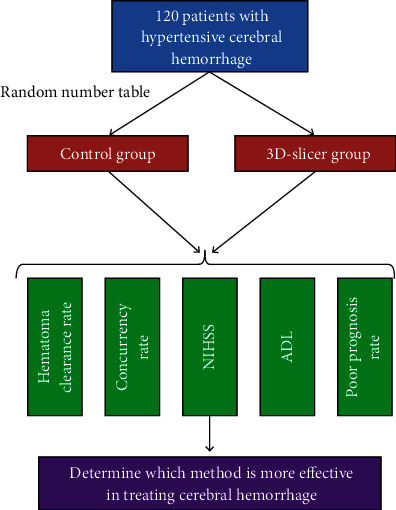
The overall structure of the full text.

The overall structure of this study is shown in Figure 1. A controlled experiment is used to verify the efficacy of the 3D-slicer software in assisting neuroendoscopic surgery in the treatment of hypertensive cerebral hemorrhage.

### 2.2. Sample Data

The study period was from October 2017 to July 2020. 120 patients with hypertensive cerebral hemorrhage who came to our hospital were selected and randomly divided into control group and 3D-slicer group with 60 cases in accordance with the random number table method.

**Figure 2:**
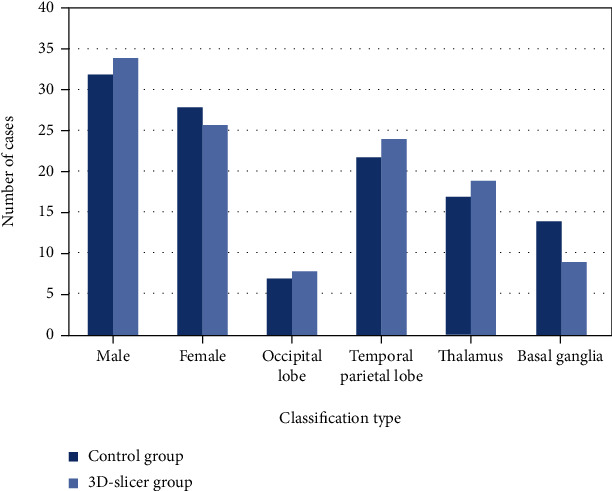
The distribution of men and women in the two groups and the location of cerebral hemorrhage.

The ratio of men to women in the control group and the 3D-slicer group and the location of cerebral hemorrhage are shown in Figure 2. There was no statistically significant difference between the two groups of patients in general data such as gender, age, and bleeding site (*P* > 0.05). The age distribution and average age are shown in Table 1.

### 2.3. Selection Criteria

The inclusion criteria were as follows: ① all were diagnosed by head CT; ② none of them could undergo craniotomy; and ③ all met the indications for puncture and drainage surgery.

The exclusion criteria were ① patients with cerebral herniation; ② patients with severe coma and unconsciousness; ③ patients with cerebral hemorrhage caused by other diseases; and ④ patients with unstable vital signs.

### 2.4. Study Population

#### 2.4.1. Control Group

In neural endoscopic surgery for hypertensive cerebral hemorrhage using conventional chord-to-body surface positioning, select the largest slice of hematoma, measure the linear distance from the puncture point to the anterior or posterior midpoint, that is, the chord distance, and convert it to the cranial body surface. In this research, we are to determine the puncture point. During the operation, the puncture is perpendicular to the center of the false hematoma projection, and the puncture is withdrawn after reaching the estimated depth. Through the transparent working sheath, the endoscope is introduced to remove the hematoma.

#### 2.4.2. 3D-Slicer Group

**Figure 3:**
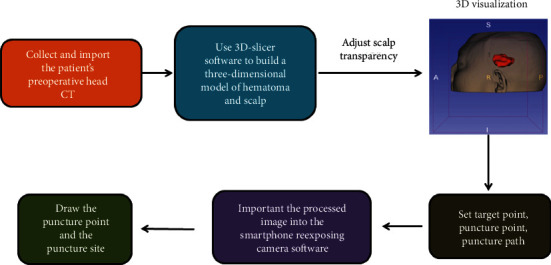
Specific steps assisted by the 3D-slicer software.

In order to evaluate the method of the 3D-slicer technique, we deliberately simplified the method to the flowchart shown in Figure 3. We specifically introduce the subsequent puncture treatment. After routine anesthesia, a disposable cranial cone with a diameter of 4 mm is used to penetrate the scalp tissue, skull, and dura of the patient, and a soft channel drainage tube with a needle core is inserted according to the set puncture route. After reaching the ideal depth, remove the needle core, and you can see the dark red blood flowing out. Finally, we aspirate the hematoma with a needle tube, suture and fix the drainage tube, and connect a sterile drainage bag.

### 2.5. Evaluation Index

#### 2.5.1. Hematoma Clearance Rate

The operation time, intraoperative blood loss, hospitalization time, and hematoma clearance rate were calculated for the two groups of patients. Head CT was used to calculate the hematoma clearance rate. The calculation method is as shown in Equation (1).

(1) $$\begin{array}{c} H=\frac{P-V_{3}}{P}\times 100\% \end{array}$$

In Equation (1), *H* represents the hematoma clearance rate, *P* represents the hematoma volume before operation, and *V* represents the hematoma volume 3 days after operation.

#### 2.5.2. Complications

In this paper, we mainly observe and count the occurrence of postoperative infection, rebleeding, cerebral infarction, and stress ulcer and calculate the incidence of complications. The calculation method is shown in Equation (2).

(2) $$\begin{array}{c} \text{C}=\frac{I+R+\text{CR}+\text{SC}}{T}\times 100\% \end{array}$$

In Equation (2), *C* represents the complications, *I* represent the infection, *R* represents the rebleeding, CR represents the cerebral infarction, SC represents the stress ulcer, and *T* represents the total number of cases.

#### 2.5.3. Neurological Function Evaluation

The National Institutes of Health Stroke Scale (NIHSS) [21, 22] was used to evaluate the neurological function of patients before and 3 and 6 months after the operation. The total score was 42 points. The higher the score represents the more severe the neurological deficit.

#### 2.5.4. Evaluation of Daily Living Ability

The ability of daily living (ADL) [23, 24] was used to evaluate the ability of daily living of the two groups of patients before and after 3 and 6 months. The total score was 100 points. The higher the score represents the stronger the ability of daily living.

#### 2.5.5. Poor Prognosis

The two groups were followed up for 3 months to compare the poor prognosis of the two groups, and the Glasgow Outcome Scale (GOS) [25, 26] was used to evaluate. One is divided into death from illness (I); 2 is divided into plant survival (PS); 3 is divided into severe disability (SM) which is conscious and needs care in daily life; 4 points in mild disability but can live independently; 5 is divided into good recovery and normal life accompanied by mild defect. The calculation of poor prognosis (*P*) is shown in Equation (3).

(3) $$\begin{array}{c} P=\frac{I+\text{PS}+\text{SM}}{T} \end{array}$$

In Equation (3), *T* represents the total number of cases.

### 2.6. Statistical Methods

We use SPSS 21.0 statistical software for the analysis. Measurement data with normal distribution and uniform variance are expressed as mean ± standard deviation. The comparison between groups adopts independent sample *t* test. The comparison of grade grouping data adopts group design and two sample comparison. The rank sum test (Wilcoxon two-sample comparison method) is based on test level: *α* = 0.05, two-sided test.

## 3. Results

### 3.1. Head CT

**Figure 4:**
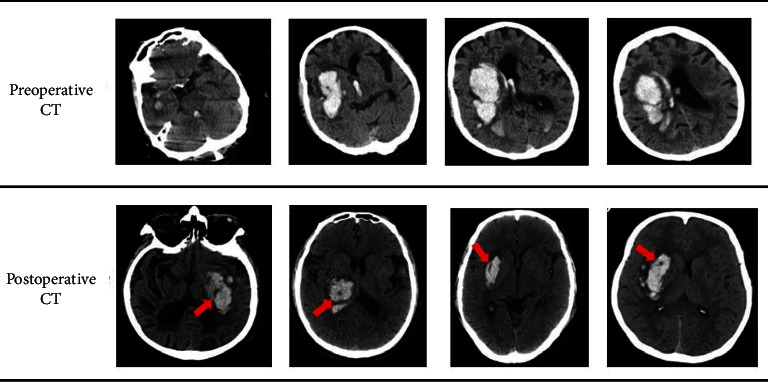
Head CT before and after surgery.

Figure 4 shows the head CT before and after the operation. The red arrow points to the hematoma.

It can be seen from Figure 4 that through the 3D-slicer software, the hematoma can be located more efficiently and the therapeutic effect of cerebral hemorrhage can be improved.

### 3.2. Comparison of Hematoma Clearance Rate

Table 2 shows the comparison of operation time, intraoperative blood loss, hospital stay, and hematoma clearance rate between the two groups.

It can be seen from Table 2 that the 3D-slicer group is stronger in terms of operation time, intraoperative blood loss, hospitalization time, and hematoma clearance rate. Therefore, the use of 3D-slicer to treat hypertensive cerebral hemorrhage is worthy of clinical promotion.

### 3.3. Complications

**Figure 5:**
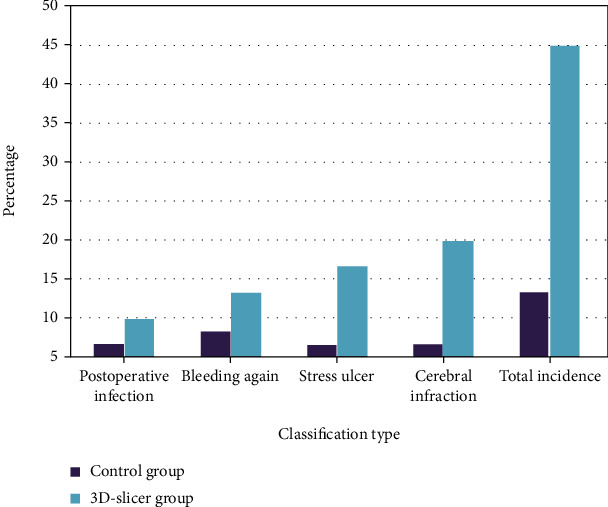
Comparison of complications between the two groups.

The overall proportion of postoperative infection, rebleeding, cerebral infarction, and stress ulcer in the 3D-slicer group was significantly lower than that in the control group, as shown in Figure 5.

### 3.4. Evaluation of the Nerve Function and Ability of Daily Living

There was no statistically significant difference between the two groups of patients in the preoperative nerve function and ability of daily living; 3 and 6 months after surgery, the nerve function and ability of daily living of the two groups were better than those before operation, and the 3D-slicer group had neurological function. The improvement of daily living ability was higher than that of the control group, and the difference was statistically significant, as shown in Table 3.

### 3.5. Comparison of Poor Prognosis

**Figure 6:**
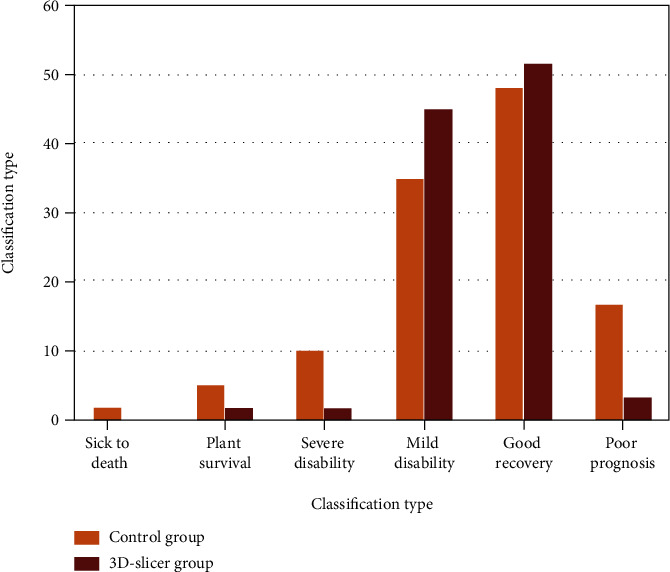
Comparison of poor prognosis.

The incidence of poor prognosis in the 3D-slicer group was lower than that in the control group, and the difference was statistically significant (*P* < 0.05), as shown in Figure 6.

## 4. Discussion

At present, CT equipment has been quite popular in domestic medical units, especially the emergence of spiral CT. Volume scanning can provide more abundant internal information of the human body [27], but the limited two-dimensional information is still used. With the development of computer technology, many image postprocessing software continue to emerge.

The 3D-slicer software allows us to use the original DICOM format data of CT and MRI to reconstruct the tissues and organs of the human body. It is compatible with Windows, Lunix, and MAC operating systems and can run smoothly on personal computers with relatively simple operation.

At present, the surgical treatment of cerebral hemorrhage is basically to remove hematoma under the microscope. The advantage of removing hematoma under a craniotomy microscope is thorough removal, but the operation time is longer and the amount of bleeding is high, which requires higher requirements for the surgeon and requires strong microneurosurgical skills [28, 29]. Because the surgical trauma is large, the patient is bedridden for a long time, there are many complications, and the prognosis is poor, which seriously increases the medical burden. Under the guidance of the concept of precision neurosurgery in the early stage, we learned and introduced the minimally invasive method of endoscopic removal. This technique can be used to open the craniotomy with a small incision, insert the puncture device to the center of the hematoma, guide the introducer to the ideal position, introduce the endoscope and suction device, and suck the hematoma.

The 3D-slicer software technology is widely used in the treatment of hypertensive cerebral hemorrhage. It assists neuroendoscopic minimally invasive surgery to effectively guarantee the quality of treatment and reduce complications. Endoscopic minimally invasive surgery has the advantages of fewer traumas, shorter operation time, less blood loss, higher hematoma clearance, less damage to the brain nerves of the patient, and fewer complications.

The 3D-slicer software may also be applied to study bone structure that is heavily influenced by the material properties on CT due to its different bone properties, which could be measured by various techniques [30, 31]. The implementation of a predictive control method [32] onto the diagnosis of patients using the 3D-slicer would enhance the effectiveness and efficiency of the technique.

## 5. Conclusion

Through the comparison of experimental data, the 3D-slicer group is better than the control group in various indicators, and the difference between the groups is statistically significant (*P* < 0.05). Compared with the control group, the 3D-slicer group has lower complications and hematoma. The clearance rate is slightly higher, the recovery of nerve function and daily living ability before and after surgery is better, and the incidence of poor prognosis is lower.

In summary, the 3D-slicer software-assisted endoscopic treatment for patients with hypertensive intracerebral hemorrhage has a better hematoma clearance effect, which is beneficial to the patient's early recovery and reduces the damage to the patient's brain nerve.

## Figures and Tables

**Table 1:** Age distribution and average age.

Group	Age range	Average age
Control group	43-82	61.2 ± 1.7
3D-slicer group	45-83	62.1 ± 1.2

**Table 2:** Comparison of operation time, intraoperative blood loss, hospital stay, and hematoma clearance rate.

Type	Control group	3D-slicer group
Operation time (min)	91.4 ± 10.8	62.0 ± 8.9
Intraoperative blood loss (ml)	110.2 ± 11.8	52.0 ± 12.4
Hospital stay (day)	23.4 ± 2.3	17.5 ± 2.4
Hematoma clearance rate (%)	77.9	97.8
t	7.458	7.846
P	0.007	0.006

**Table 3:** Comparison of nerve function and ability of daily living.

Group	Nerve function			Ability of daily living		
	Preoperative	3 hours after surgery	6 hours after surgery	Preoperative	3 hours after surgery	6 hours after surgery
Control group	24.57	18.47	15.22	50.76	71.84	82.33
3D-slicer group	23.98	13.41	8.75	51.33	80.62	92.74

## Data Availability

Data is available upon request from the corresponding author.
